# Exploring the Transition to Fatherhood: Feasibility Study Using Social Media and Machine Learning

**DOI:** 10.2196/12371

**Published:** 2018-11-27

**Authors:** Samantha J Teague, Adrian BR Shatte

**Affiliations:** 1 Centre for Social and Early Emotional Development School of Psychology Deakin University Burwood Australia; 2 School of Science, Engineering and Information Technology Federation University Berwick Australia

**Keywords:** parenting, perinatal care, fathers, social media, parent-child relations, infodemiology, unsupervised machine learning

## Abstract

**Background:**

Fathers’ experiences across the transition to parenthood are underreported in the literature. Social media offers the potential to capture fathers’ experiences in real time and at scale while also removing the barriers that fathers typically face in participating in research and clinical care.

**Objective:**

This study aimed to assess the feasibility of using social media data to map the discussion topics of fathers across the fatherhood transition.

**Methods:**

Discussion threads from two Web-based parenting communities, r/Daddit and r/PreDaddit from the social media platform Reddit, were collected over a 2-week period, resulting in 1980 discussion threads contributed to by 5853 unique users. An unsupervised machine learning algorithm was then implemented to group discussion threads into topics within each community and across a combined collection of all discussion threads.

**Results:**

Results demonstrated that men use Web-based communities to share the joys and challenges of the fatherhood experience. Minimal overlap in discussions was found between the 2 communities, indicating that distinct conversations are held on each forum. A range of social support techniques was demonstrated, with conversations characterized by encouragement, humor, and experience-based advice.

**Conclusions:**

This study demonstrates that rich data on fathers’ experiences can be sourced from social media and analyzed rapidly using automated techniques, providing an additional tool for researchers exploring fatherhood.

## Introduction

### Background

The transition to parenthood is a critical period for fathers and their families alike. The changing role from individual to partner to parent across the prenatal, perinatal, and postnatal periods requires men to undergo significant psychological transition [1]. The social and emotional changes that expectant fathers experience across the perinatal period are associated with increased risk of mental ill health in fathers [1,2]. An estimated 1 in 10 fathers experience depression across the perinatal period, and a further 2.4% to 18% of fathers may experience anxiety disorders [2,3]. The impact of poor mental health in fathers across the fatherhood transition can affect the entire family, with implications for the mental health of mothers and their combined parenting relationship [4] as well as implications for the development and well-being of children [5].

Despite the importance of parenting transitions for the well-being of fathers and their families, research exploring this topic is limited. Reviews to date have repeatedly noted the paucity of research available for synthesis [1,6,7], particularly when compared with studies exploring the transition to motherhood [1,2]. A key reason for the underreporting of fathers’ experiences in the literature is the difficulty in reaching and engaging fathers in health research and practice [8,9]. Reasons for this difficulty reported in the literature include the perceived disinterest of fathers, the reluctance of mothers to involve fathers in perinatal care, stereotypes of men being incompetent or absent when it comes to caregiving, and fathers’ own lack of help seeking [9,10].

Furthermore, research that does explore fatherhood transitions is typically limited by a number of methodological issues, as identified by a recent systematic review [6]. First, much of the research exploring fathers’ transitions relies on mothers’ reports of fathers’ experiences rather than direct assessment of fathers themselves, with the validity of mothers’ reports questionable [11,12]. Second, data collection periods typically capture fathers’ experiences from the birth of their child onward, failing to capture fathers’ prenatal experiences. Furthermore, research is typically focused on fathers’ role in the marital relationship, despite transitions to parenthood being conceptualized as a complex, multidimensional process. Finally, research is typically completed using small sample sizes, assumedly because of the difficulty in recruitment and retention of fathers in research and practical limitations of the qualitative research methods typically employed. These difficulties in adequately capturing fathers’ experiences might have flow-on effects into treatment services, where fathers are reportedly difficult to engage and treatments are likely to be ineffective [9].

Such methodological difficulties highlight the need for innovation in the way we engage men in research exploring their transition to parenthood. One promising area is through social media. Social media platforms offer an opportunity for researchers to passively track the health and well-being of the public at scale, regardless of their geographic location [13]. The natural formation of group discussions on social media offers researchers the opportunity to observe ecologically valid models of social support processes. It also offers the ability for researchers to capture rare in-the-moment data when people are experiencing an event rather than relying on their retrospective reporting (eg, via surveys or interviews).

Social media is a particularly promising avenue for fatherhood research. Although fathers are difficult to engage through traditional support settings, evidence suggests that electronic health strategies might be a more effective avenue at reaching and engaging fathers [14,15]. Fathers themselves report using the internet as a key source of information about pregnancy, childbirth, and postnatal care [16], and social media is used by fathers particularly for social support regarding their fatherhood role [17]. Research on fathers using social media has explored topics such as health care parental support [8], young fathers’ experiences (fathers aged under 22 years) [18], the nature of social support provided through social media to fathers [19,20], and differences in the parenting roles of mothers and fathers [21].

Very little research has examined the feasibility of using social media data to explore fatherhood transitions specifically. A study by StGeorge and Fletcher [19,22] examined discussions between 23 current and expectant fathers in an Australian online forum over an 18-month period. Discussions converged onto 4 overarching themes: fathers’ feelings of isolation because of the low support fathers receive, fathers’ practices in balancing work and family life, social opinions on parenting issues such as breastfeeding, and announcements such as birth announcements and physical meet-ups. Although this study demonstrated that fathers are willing to discuss their experiences of the transition to fatherhood with other fathers on the Web, further research is needed to examine whether these results are generalizable to fathers’ discussions on larger social media forums, with more diverse groups of fathers.

More recently, new techniques such as machine learning have emerged to explore social media data at scale. Machine learning refers to a subset of artificial intelligence algorithms that learn and increase in performance as new data are analyzed [23]. There are 3 primary types of machine learning techniques: supervised learning, in which data with known labels are used to create predictive models; unsupervised learning, in which mathematical techniques are used to cluster and distinguish patterns from uncategorized data; and semisupervised learning, in which both labeled and unlabeled datasets are used to make predictions. Machine learning has already been applied to many areas of parenting on social media such as predicting postpartum depression from Facebook posts [24] and analyzing vaccination discussions on mother forums [25]. Within the fatherhood space, Ammari et al [21] explored the influence of gender on parental roles by applying machine learning to both mother and father discussion forums. Using Latent Dirichlet Allocation (an unsupervised machine learning technique), the authors found that machine learning was useful for mapping parenting discussions on social media. This study demonstrates that fathers are very active on social media sites (specifically the Reddit platform), offering the opportunity for new research avenues and additional machine learning techniques to be explored with fatherhood discussions on social media.

### Objectives

This study, therefore, aimed to assess the feasibility of using social media data to explore changes in the discussion topics as men transition to fatherhood. Specifically, we aimed to map the discussion topics held between both expectant and current fathers on the international forum *Reddit*. We expected to observe fathers discussing similar issues to that reported in previous research, such as work-family life balance, parenting practices, and difficulties engaging with health professionals, within a framework of positive social support. We then aimed to compare and contrast these discussions between expectant and current fathers, expecting to find overlapping topics such as support and issues with spouses, sleep difficulties, and fatherhood anxiety.

## Methods

### Datasets

We explored topics of discussion from two publicly accessible Web-based parenting communities within the Web forum Reddit. As of April 2018, Reddit is the fifth most popular website in the United States (18th globally), with an average of 274 million unique users per month [26]. Users are typically college-educated males from the United States, aged under 35 years [26,27]. Reddit users can create forums based on topics of interest (called subreddits) to share and discuss related content with other users who subscribe to their own topics of interest to form special interest communities. Communities are self-moderated using an in-built voting system; *upvotes* increase a post’s visibility, whereas *downvotes* decrease a post’s visibility. Posts (ie, top-level discussion topics or individual comments in a discussion thread) are generated a total score by subtracting downvotes from upvotes.

There are several distinct subreddits that focus on parenting within Reddit, including r/Parenting (for general discussions on parenting), r/SingleParents (specifically for single parents), and r/Mommit (a place for mothers to discuss parenting). As the motivation of this study was to explore issues in the transition to fatherhood, we focused specifically on the 2 most popular subreddits for fatherhood, including r/Daddit (79,019 subscribers as of April 2018) and r/PreDaddit (17,923 subscribers as of April 2018), which are targeted to new fathers during pregnancy. Using the Reddit application programming interface (the official set of tools that provide third-party software with access to Reddit data), we first downloaded the most recent discussion threads (eg, original posts and their associated comments combined) from both of these subreddits, which are limited to a total of the most 1000 recent posts per subreddit. We then continued to automatically collect all new discussion threads from both of these subreddits over the proceeding 2-week period (March 23, 2018, to April 06, 2018). We also collected additional metadata, including post and comment scores (as voted by users), and anonymous user IDs to track participation across both subreddits by individual users.

### Analysis

Several steps were taken to prepare the data for analysis using the Python Natural Language Toolkit (version 3.2.5) and scikit-learn (version 0.19.1). First, original posts and associated comments were grouped together to form single documents for analysis. We then cleaned the data by removing punctuation, stop words, and low- and high-frequency terms, followed by stemming (reducing words with similar meanings to their base form) and tokenization (splitting the posts into individual words while removing most punctuation). Documents with less than 50 total characters remaining were then removed to help improve algorithm accuracy. Finally, each document was vectorized (converting the text-based documents to numerical vectors) using term frequency-inverse document frequency (a method that weighs each word in a document based on the number of times it appears, offset by the number of documents in which that word appears).

Topic modeling was then performed using the machine learning algorithm *k*-means clustering. The *k*-means clustering technique is a widely used unsupervised clustering algorithm that can create clusters of documents that are similar to one another and dissimilar from the documents that are in other clusters [28]. One of the primary benefits of clustering techniques is that they are exploratory in nature, in the sense that they do not use predefined labels to group data [28]. We used the *k*-means clustering algorithm in sci-kit learn to analyze the discussion content from both subreddits as well as a combined corpus (the entire collection of posts). The clustering was performed for different values of *k* ranging from 5 to 15. After manually analyzing the results of clustering on each value of *k*, we settled on 9 clusters as the best value to represent the distinct discussion topics for each subreddit and the combined corpus.

We then further grouped the 9 discussion topics into broader emergent categories by using an open coding process as follows. First, the posts within each discussion topic were sorted by proximity to the center of the cluster, as more central posts best represent the features used by the algorithm to cluster the topic. Next, posts were sorted by user score to determine which posts were most important to the community. For both sorting methods, the authors independently read the posts in order and proposed a category theme. Discrepancies were discussed, and categories were revised until both authors agreed that the category label described the primary themes. As the primary aim of this study was to assess the feasibility of machine learning to map discussion topics of parenting in social media data, we chose to focus on simply labeling the topics rather than providing nuanced analysis on discussion content.

Finally, to compare the similarity and diversity of each topic between groups, Jaccard similarity coefficients were calculated. The Jaccard similarity coefficient is a common technique used to compare similarity and diversity of sets [29]. It is calculated by dividing the intersection of 2 sets by their union and multiplying the result by 100 to obtain a score. A score of 0 indicates that there is a large amount of diversity and difference between 2 topics, whereas a score closer to 1 indicates that these topics contain more similar words.

Results are presented by first mapping discussion topics across the fatherhood transition (ie, using both the r/PreDaddit and r/Daddit forums), then mapping the discussions of expectant and current fathers separately, and finally by comparing the discussions of expectant and current fathers.

### Ethics in Data Collection on Social Media

Although the public nature of social media forums such as Reddit means that formal human research ethics approval is not required to use the data, there are other important ethical considerations that should be adhered to. Previous research has indicated that although contributors to public forums acknowledge a certain degree of open access, they still may not post with the intention of public visibility and still seek privacy where possible, for example, by using pseudonyms [30]. In comparison with social media networks such as Facebook and Twitter, which are linked to the true identity of each user, members of Reddit typically select pseudonymous usernames. Due to the nature of discussion topics (eg, topics on children), we deidentified any comments that included names and places.

## Results

### Discussion Topics Across the Fatherhood Transition

The final cleaned dataset resulted in 1980 discussion threads, which were contributed to by 5655 unique users, totaling over 765,000 words across a 6-month period (November 12, 2017, to April 06, 2018). The 9 discussion topics were grouped within 3 emergent categories: (1) Expectant Fathers’ Milestones, including *Pregnancy Milestones*, *Birth Announcements*, and *First-time Fathers*; (2) Fathers’ Practices, including the topics of *Growing Up*, *Cute Pictures*, and *Paternal Bonding*; and (3) Fatherhood Challenges, covering the topics *Struggles*, *Budgets and Purchases*, and *Sleep*.

Table 1 displays the descriptive statistics of each discussion topic. Overall, the Struggles discussion topic contained the largest number of posts, with 436 discussion threads and 4380 replies contributed by 2702 users. Unsurprisingly, the Struggles discussion topic also achieved the highest total user score (the combined score of all posts within the topic, as voted by users). The smallest discussion topic was Birth Announcements, which contained 57 discussion threads with 255 replies, contributed by 250 users.

#### Expectant Fathers’ Milestones

The first emergent category, Expectant Fathers’ Milestones, captured expectant and new fathers’ experiences across the perinatal period. Most of the discussions across the category were celebrations of major events and milestones, such as *graduating* to fatherhood, with replies of encouragement and congratulations from the wider community:

Original post:

Graduated! Baby finally showed up 8 days late and 34 hours of labor from my rock star wife. 7lbs 10oz!

Reply:

Currently in the hospital at 5 min contractions we are 2 days past due date so hoping this is it!!! Congrats man!

Birth Announcement

The community also helped to bring greater awareness of the challenges involved in the perinatal period, particularly around difficulties in conceiving and carrying the infant to term, for example:

Miscarriages are actually quite common. They just are not talked about much. I wish people would talk about them more to help other people. My wife had an ectopic pregnancy and lost one of her tubes. Since then, we now have an almost 4yo and a 6mo. A friend of ours had 3 miscarriages before a successful pregnancy. Good luck [original poster], and let the rollercoaster ride begin!

Pregnancy Milestones

The experiences of first-time fathers were particularly supported by the community, with posts in this category receiving an average score of 1702 (calculated by dividing the total user score of the category by the number of individual posts in the category). First-time father discussions were characterized by support and humor, for example:

Original post:

Today I learned to be a dad for the first time.

Reply 1:

Being dad is a lifelong lesson, may you always be open to learning. Enjoy the adventure dad!

Reply 2:

Nah, you just read the jacket cover and got excited about what comes next. Wait until you get to Chapter One, “Oh my God the poop has started to smell.” The real good reads are the chapters “I was just pee’d on” and “I think I got throw up in my mouth.” Don’t buy the Cliff Notes. Congratulations on spending the rest of your life trying to hide the fact that you have no idea what your doing from your little one. You’re one of us now.

First-time Fathers

**Table 1:** Emergent categories in the discussion topics of fathers across the fatherhood transition.

Topic		Discussion threads, n	Replies, n	Total user score	Word count
**Expectant Fathers’ Milestones**					
	Pregnancy milestones (users: n=740)	235	1078	13,292	51,916
	Birth announcement (n=250)	57	255	6086	3754
	First-time fathers (n=803)	152	945	25,876	12,414
**Fathers’ Practices**					
	Growing up (n=1856)	446	2474	45,920	1,31,526
	Cute pictures (n=1040)	316	1074	29,865	26,667
	Paternal bonding (n=831)	133	871	28,486	16,799
**Fatherhood Challenges**					
	Struggles (n=2702)	436	4380	57,976	4,31,415
	Budgets and purchases (n=503)	123	615	3490	61,126
	Sleep (n=444)	82	470	7867	29,651

#### Fathers’ Practices

While Expectant Fathers’ Milestones focused on perinatal experiences, Fathers’ Practices instead captured fathers’ discussions on activities and feelings across their child’s early development. The *Cute Pictures* topic involved fathers’ sharing photos of their children and family with humorous captions, for example:

This one just turned 6 months, and is now pulling herself up and crushing furniture, trying to walk. She wants to chase her big sister around. She’s gonna kill me. At least she’s cute!

Cute pictures

The *Growing Up* topic included milestones in children’s development as well as sharing stories of children’s growing hobbies, interests, and personalities. As with other topics, fathers’ responses were characterized by sharing their own similar experiences and humor, for example:

Original post:

Just need the support. I am out of town for work, and I missed my sons first word. He said “hi” to my wife...

Reply:

That sucks! There’ll be many firsts. This one feels big, but the fact you weren’t present doesn’t mean you’re doing a bad job. Chin up. I got a first yesterday: first time he’s said “i don’t love you, dada!”

Growing up

Finally, the Paternal Bonding topic included discussions where fathers shared stories and photos of activities between themselves and their children, the emotions they felt about their relationship with their child, and their feelings about being a father more generally, for example:

Entered our car in the race yesterday with a lack of high hopes. 2nd place out of 65!!!! my son [the child] (7) was so happy he cried. I feel like I did something right!

Paternal Bonding

#### Fatherhood Challenges

The final semantic category that emerged from the combined discussions of expectant and current fathers was Fatherhood Challenges. This category included discussions on fathers’ *Struggles*, *Budgets and Purchases*, and *Sleep*.

The topic *Struggles* covered a variety of difficulties that fathers were facing, with fathers also seeking support from their Reddit community. Examples of discussion thread topics included coping with children’s diagnoses of serious medical conditions; difficulties in adapting to the *father* role; difficulties in relationships with partners and coparents; struggles with depression, anxiety, and other mental health conditions; and dealing with the loss of an infant. The community was strongly supportive of posts within the *Struggles* category, with the average post receiving approximately 10 replies, for example:

To all the Dads to be that are struggling; coming to grips with being a parent, dealing with an unreasonable hormonal partner, struggling to be loyal when not having your needs met, or even considered, struggling post-partum, remember, one day your kid will look at you and smile. Hang in there.

Struggles

Budgets and Purchases covered discussions about the cost of items, whether the items were necessary, and recommendations for products. Discussions were typically focused on expectant fathers and new fathers asking for recommendations and advice about the etiquette of baby shower registries and products for infants, particularly car seats and strollers, for example:

Original post:

Cheap vs Expensive baby stuff: whats the actual difference? [...] Just trying to wrap my head around how to research and select. [...]. its not that i cant afford it, its that I’d rather save the money and put the rest into funds for future expensive events like college. and food.

Reply:

This is a great question. I have no idea how to shop for anything either, so thank you for asking this!

Budgets and Purchases

The topic of sleep emerged as a distinct discussion topic across the combined fatherhood transition. Discussions focused on fathers’ struggles with their own sleep quality and fatigue, issues in helping infants and young children with sleep, and queries about sleep routines. Importantly, fathers typically recognized that sleep disturbances were a normal part of life after the birth of a child, for example:

3 days in from the birth of my little girl. Sleep has become a thing of the past now. Had my little princess on my chest since two in the morning to allow my wife to sleep. Just. So. Tired. But my little girl is just so perfect.

Sleep

### Discussion Topics of Expectant Fathers

Discussions collected from r/PreDaddit included 1031 discussion threads consisting of 446,060 words. These discussions were contributed to by 2366 unique users across a 6-month time frame (November 2017 to April 2018). A total of 9 discussion categories were identified (see Table 2), including *Nesting*, *Pregnancy Complications*, *Budgets and Purchases, Standby!*, *Birth Announcements, Gender Announcements, Pregnancy Milestones, Fertility Issues*, and *Pregnancy Announcements*. These 9 categories were further synthesized into 3 semantic categories: *Announcements, Preparing for Fatherhood*, and *Pregnancy Events,* which are discussed below.

Table 2 displays the descriptive statistics of each discussion topic. Overall, the Pregnancy Complications discussion topic contained the largest number of posts, with 169 discussion threads and 1628 replies contributed by 984 users. The Birth Announcements discussion topic achieved the highest total user score, despite having only a moderate number of posts. The smallest discussion topic was Pregnancy Announcements, which contained 33 discussion threads with 111 replies, contributed by 127 users.

**Table 2:** Emergent categories in the discussion topics of expectant fathers in r/PreDaddit.

Topic		Discussion threads, n	Replies, n	Total user score	Word count
**Announcements**					
	Birth announcement (users: n=432)	103	541	12,974	6088
	Gender announcement (n=401)	100	459	5966	15,627
	Standby! (n=343)	111	389	4321	23,913
	Pregnancy announcements (n=127)	33	111	2278	2849
**Preparing for fatherhood**					
	Nesting (n=828)	248	1227	9695	1,08,362
	Budgets and purchases (n=575)	133	813	4921	81,941
**Pregnancy events**					
	Pregnancy complications (n=984)	169	1628	12,281	1,82,278
	Pregnancy milestones (n=313)	85	339	3015	18,179
	Fertility issues (n=242)	49	247	4157	6823

#### Announcements

The first emergent category that was identified from the r/PreDaddit discussions was *Announcements*. A common type of post identified in the forum was expectant fathers sharing their excitement along the timeline of conception to birth, including the announcements for pregnancy (*Pregnancy Announcement*), gender (*Gender Announcement*), imminent birth (*Standby!*), and birth (*Birth Announcement*). Usually, the original posts were short in word count, but included highly positive language with themes of happiness and joy, for example:

She’s getting induced tonight. Wish us luck! We’re having our very first baby tonight. I can hardly contain my excitement. Wish us luck Reddit. I will report back when she’s here! Update: she’s still in labor, probably going to be a long one at this rate but she’s doing amazing! Thank you all for your support!

Standby!

Replies to such posts were also extremely encouraging and congratulatory in nature, for example:

Congrats dude! Isn’t it such a relief to finally not have to hide this major life changing news!? Enjoy the tidal wave of love and excitement from loved ones!

Pregnancy Announcement

After the birth of their child, expectant fathers sent their farewells to the r/PreDaddit forum as they *graduated* to the forum for current fathers, r/Daddit:

Graduated! Thank you so much to the kind strangers on here for all of the advice. r/Daddit, here I come.

Birth Announcement

#### Preparing for Fatherhood

Another theme that emerged from the identified categories was the preparation for fatherhood, including preparing the home for the incoming baby (*Nesting*), as well as discussions and recommendations on products and budgeting for the additional costs of having a child (*Budgets and Purchases*). *Nesting* discussions typically included expectant fathers sharing their excitement about the impending birth by showing pictures of their new nursery:

[Baby] will be here in 13 days or less! Here’s his nursery wall I made.

Nesting

*Nesting* also included discussions preparing for changes to the family unit, including pets:

I don’t graduate until 8th September but already trying to get things in place and was wondering how you’ve introduced family dogs to the new borns. We have a very boisterous red setter/lab mix and I want to get all the advice possible I can to handle the situation well. I know first off that I need to be as calm as possible about as dog will react to any feelings of apprehension but any advice welcome.

Nesting

The *Budgets and Purchases* topic included discussions between expectant fathers seeking suggestions on baby products, for example:

Now that it’s becoming really real for us, I was just curious for predads that are further along and those that have graduated, what was your first purchase for the baby? When did you and your partner begin making purchases to prepare for the new family member? I’m antsy to get the car seat and nursery rocking chair figured out asap, even though those might not be the biggest priority -_-

Budgets and Purchases

*Budgets and Purchases* also included longer-term planning by expectant fathers about the upcoming costs associated with having children:

My wife just found out that she is pregnant (5 weeks- we were trying)! Hooray! We are very excited and know we will be “OK” with getting things ready for the baby - the only thing we’re both freaking out about is childcare after birth...We both work fulltime (she works 40-80hrs/week) and both currently work weekends or sometimes her schedule is random. She will get a month or two of maternity leave. After that...? We don’t have any family support anywhere near us, so I guess it is either a nanny or childcare center. Beyond the obvious freak-outs about handing off your 6-8 week year old to a stranger, we’re wondering how the hell we afford something like this?

Budgets and Purchases

#### Pregnancy Events

The final emergent category from the expectant father forum was *Pregnancy Events*, which included posts about medical or miscarriage issues during pregnancy (*Pregnancy Complications*), updates about the progress of the mother and baby during the pregnancy timeline (*Pregnancy Milestones*), and posts about issues faced when conceiving a baby (*Fertility Issues*).

*Pregnancy Complications* included discussions on issues within the pregnancy, including miscarriages, genetic screening results, and other related issues. This category included posts about grief or shock over issues with the baby, for example:

No heartbeat for twins at 8 weeks. Devastated.

Pregnancy Complications

Despite the themes of loss and heartbreak that characterized most original posts in this category, there were also posts containing messages of support to encourage other members to keep fighting despite such setbacks, for example:

To all the Dads-to-be that are struggling; coming to grips with being a parent, dealing with an unreasonable hormonal partner, struggling to be loyal when not having your needs met, or even considered, struggling post-partum, remember, one day your kid will look at you and smile. Hang in there.

Pregnancy Complications

Another interesting theme that was uncovered in the *Pregnancy Complications* category was fathers discussing their own challenges during pregnancy, reacting to physical and emotional changes of their wife, for example:

I knew that she was pregnant. I knew her body was changing and that she needed special care and attention. But even though I knew there was a baby in there, I didn’t really get it until the baby was born. I’ve talked with many of my friends who had the same experience. Months of pregnancy, birth - here is a baby! It’s weird.

Pregnancy Complications

In comparison with *Pregnancy Complications*, the posts that were categorized as *Pregnancy Milestones* included fathers sharing updates about the progression of the pregnancy, for example:

After a threatened miscarriage at 8 weeks, we hit 12 weeks 6 days today. Baby steps, pun intended.

Pregnancy Milestones

Felt the baby kick last night.

Pregnancy Milestones

Interestingly, the posts in the *Fertility Issues* category involved fathers who had experienced difficulties in conceiving a baby, with many sharing news that they were now expectant fathers, for example:

After two miscarriages and months of fertility treatments, we are due in December.

Fertility Issues

Joining the club after 4 years of infertility.

Fertility Issues

### Discussion Topics of Current Fathers

Discussions collected from r/Daddit included 949 discussion threads consisting of 319,208 words. These discussions were contributed to by 3672 unique users across a 3-month time frame (February 2018 to April 2018). A total of 9 discussion categories were identified (see Table 3), including *Struggles, Child’s Growth and Development, Paternal Bonding, Parenting Skills, Feeding, Play, Dad Hobbies, Meet my Baby,* and *Easter*. These 9 categories were further synthesized into 3 semantic categories: *Help Seeking, Fathers’ Practices*, and *Community Building,* which are discussed below.

**Table 3:** Emergent categories in the discussion topics of current fathers in r/Daddit.

Topic		Discussion threads, n	Replies, n	Total user score	Word count
**Help seeking**					
	Struggles (users: n=2136)	277	3151	66,063	2,24,013
**Fathers’ practices**					
	Paternal bonding (n=385)	79	385	15,044	9199
	Parenting skills (n=310)	70	300	7439	9430
	Feeding (n=270)	69	273	4017	17,894
	Children’s growth and development (n=949)	238	1047	24,963	35,975
	Play (n=268)	47	246	6465	7152
	Dad hobbies (n=112)	31	97	2570	2413
**Community building**					
	Reddit, meet my baby (n=758)	124	869	32,014	12,365
	Easter (n=47)	14	40	675	767

Table 3 displays the descriptive statistics of each discussion topic. Overall, the Struggles discussion topic contained the largest number of posts, with 277 discussion threads and 3151 replies contributed by 2136 users. The Struggles discussion topic also achieved the highest total user score. The smallest discussion topic was Easter, which contained 14 discussion threads with 40 replies, contributed by 47 users.

#### Help Seeking

The first emergent theme identified within r/Daddit was *Help Seeking*. In this theme, discussions were focused on the challenges and issues fathers face in raising their child (*Struggles*), with replies consisting of other fathers providing moral support and sharing their own stories of struggling times during fatherhood. One of the primary struggles posted by fathers related to the death of their baby, evoking emotions such as sadness, heartbreak, and shock, for example:

Tonight, I had to make the hardest decision of my life. Hug your babies close tonight.

Struggles

Another common discussion point in this theme involved posts and updates by fathers whose children were battling an illness, such as leukemia or stomach problems, with those fathers reaching out to the community for moral support, for example:

Youngest admitted to the hospital last night. Just found out he has some kind of leukemia. His mom and I are still reeling.

Struggles

Apart from medical issues, fathers also discussed other issues in parenting, such as the challenges single fathers face while raising the child on their own, and day-to-day challenges such as lack of sleep because of the new baby, for example:

At 20, I had a kid with a girl, things happened, I got full custody, and very quickly I had to learn how to raise a child alone. Its really tough sometimes, but I wouldnt change it for the world.

Struggles

I thought I knew what tired was before they came.

Struggles

The comments left on threads in the *Struggles* category were primarily supportive, with users leaving positive messages of support to the original poster, for example:

Me and mine are sending our love your way. Stay strong, brother.

Struggles

#### Fathers’ Practices

Another emergent category that was identified in the r/Daddit corpus was *Fathers’ Practices*. The posts in this category included topics such as sharing bonding experiences between fathers and their children (*Paternal Bonding*), sharing questions and experiences of being a parent (*Parenting Skills*), tips and advice about feeding a new baby (*Feeding*), milestones and learning experiences with their children (*Children’s growth and development*), play time between father and son (*Play*), and fathers sharing how they like to spend their downtime while not parenting (*Dad Hobbies*).

Paternal bonding posts were primarily stories or anecdotes in which fathers described activities shared with their child, for example:

The Boy and I having a Batman sort of day.

Paternal Bonding

My 9yo son loves writing and has written several short books over the past few years. Today we took our laptops to the coffee shop to have a joint writing session. So much fun we will do it again. I may never be a great writer, but today I felt like a pretty good dad.

Paternal Bonding

Another major topic of discussion identified in this category was *Parenting Skills*, in which fathers would describe their progress in overcoming setbacks, learning new parenting skills, or seeking and sharing advice about parenting-specific experiences, for example:

Single dad with 50/50 custody. Lil guy is eight years old, so proud of him. Proud of myself over 10 months completely sober and almost two years into working on my fitness so I can be better for both of us. Life is good.

Parenting Skills

Anyone want to try and explain the two symbols under the umbrella (waterproof) to me?

Parenting Skills

Another primary theme of conversation identified in this category was *Feeding*, in which fathers would seek advice from the community about feeding their child or make humorous observations about their experiences of feeding their child, for example:

New dad, need help with feeding.

Feeding

Let’s give a happy St. Pats to all the dads drinking Guinness out of a can, washing bottles and pump parts instead of being at the pub with our mates. #adulting

Feeding

Fathers in this category also shared information on the growth and development of their child, including the reaching of developmental milestones, transferring of skills and interests between father and child, and other learning experiences encountered when growing up. The topics of discussion in this theme were grouped as *Children’s Growth and Development*, and posts consisted primarily of positive or humorous anecdotes, for example:

Little guy wanted to come to work with me today, wanted to be like dad so much he got his own uniform haha.

Children’s Growth and Development

She is just starting to learn to hold onto things, wouldn’t let go of this green bean in a super market. They wouldn’t let her leave without paying. Turns out 1 bean costs 4 cents!

Children’s Growth and Development

Congrats to the little girl losing her first tooth, here’s my youngest proudly displaying her first tooth coming in at 4 months. Relieved to find out all those fits were due to teething and not demonic possession.

Children’s Growth and Development

Replies from other fathers to posts in this theme were primarily positive as well, comprising humorous responses or fathers sharing similar anecdotes and stories from their own experiences.

In terms of *Play*, there were 2 main topics of discussion. First, fathers shared anecdotes and stories about experiences they had while playing with their child. In addition, some fathers also posted stories about their own *play* experiences, such as gaming, for example:

Howdy folks! I just recently joined your ranks and was wondering if there were any gamers out there that had any input on good games for a new dad. Basically what I’m looking for are games that are good in short bursts (not like 400 hour rpgs where you get lost if you forget where you were supposed to go), and that allow you to pause, save or quit at any time.

Play

Similarly, there were also several posts that were classified as *Dad Hobbies*, in which fathers described activities they enjoyed doing in their down time from parenting, for example:

To all the Mamas, who recognize the needs of the Daddas to go solo-watch movies about big robots fighting fish monsters & give them a nice night off of kid duties + beer money.

Dad Hobbies

Caught a bluefin trevally next to the USS MISSOURI with my little guy strapped to my chest!

Dad Hobbies

#### Community Building

The third emergent category that was identified in the r/Daddit corpus was titled *Community Building*. Posts in this category primarily involved fathers introducing their babies or children to the community (*Meet my Baby*). This type of post acted as an introduction and initiation into the community. For example:

Met my daughter at 5:04 this morning.

Meet my Baby

Wife and I welcomed our beautiful son and beautiful daughter into the world late last night!

Meet my Baby

The comments posted in response to these posts were overwhelmingly positive and congratulatory in nature, for example:

Congratulations and a warm welcome to this sub!

Meet my Baby

Congrats on your little slice of heaven!

Meet my Baby

Another small theme that appeared in this category because of the time of data collection was posts about fathers and their children celebrating the *Easter* holiday, for example:

This has been an interesting Easter, 1 month old high fevers are not fun.

Easter

### Comparison of Discussions Between Expectant and Current Fathers

**Figure 1:**
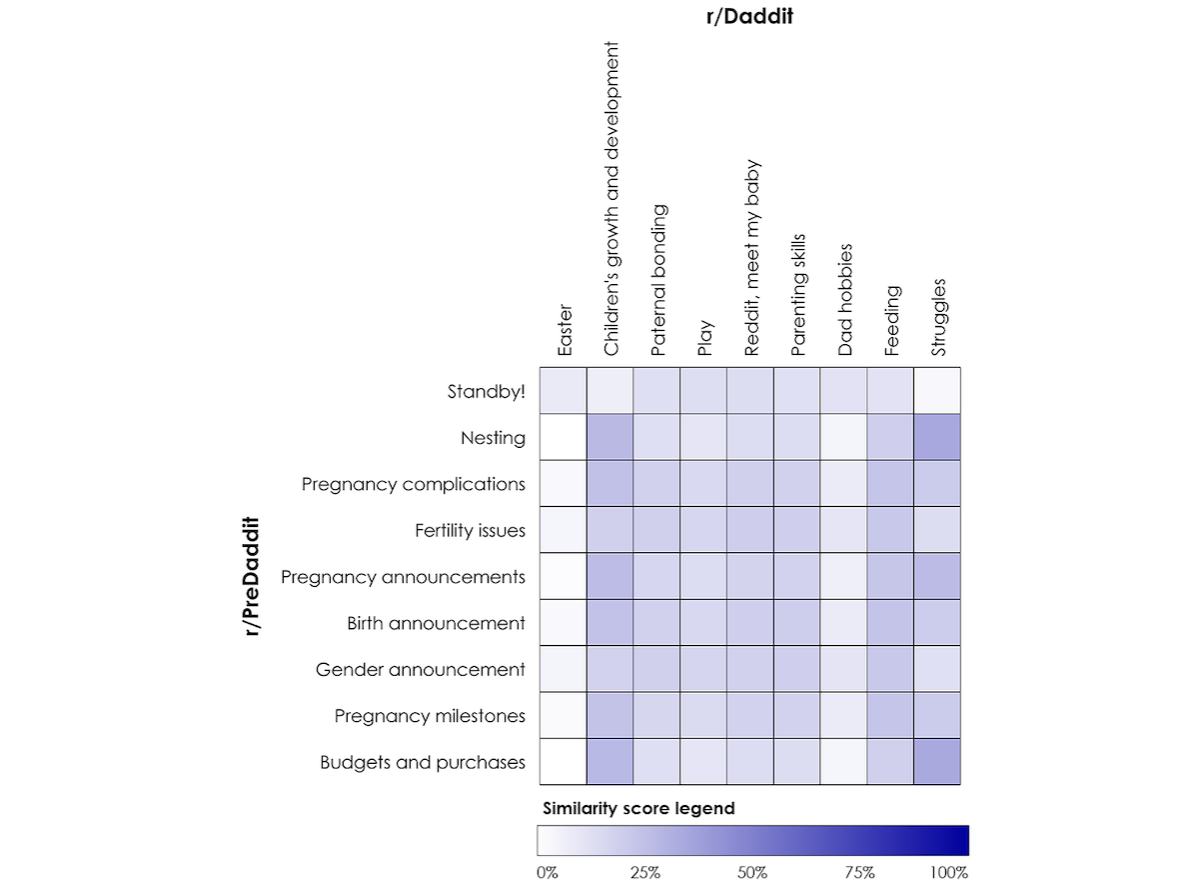
Overlap in discussion topics between expectant (r/PreDaddit) and current (r/Daddit) fathers, calculated using Jaccard scores.

A small proportion of users, 7.13% (403/5655), were found to post in both r/Daddit and r/PreDaddit. Overall, low Jaccard coefficients were observed between r/Daddit and r/PreDaddit discussion topics (<0.4), indicating high heterogeneity between the 2 subreddits. This demonstrates that although there is some overlap in themes discussed across r/Daddit and r/PreDaddit, these groups also contain distinct themes. The highest Jaccard score identified was between Nesting and Struggles (0.36), Budgets and Purchases and Struggles (0.36), and Nesting and Children’s Growth or Development (0.30). The lowest scores were observed between Standby and Struggles (0.06) and between Nesting and Dad Hobbies (0.07), and nearly all r/PreDaddit topics compared with Easter were <0.07. Figure 1 displays the overlap between topics.

## Discussion

### Principal Findings

This study aimed to assess the feasibility of using machine learning to explore the discussion topics of Web-based communities as men transition to fatherhood. This was achieved by mapping the discussion topics on 2 forums for expectant and current fathers from the social media site Reddit. In doing so, this study has demonstrated that Reddit is an ecologically valid tool for exploring current issues for men as they transition to fatherhood. Such research has important implications for the design of online social support systems for fathers and for the research methodologies used to reach and engage fathers across the perinatal period and beyond.

This study demonstrates that social media data can provide a convenient and ecologically valid method for obtaining rich information on the issues fathers face both antenatally and postnatally. As previous studies have demonstrated, the experience of fathers is often difficult to capture in formal research and support settings. Clustering the discussion topics of current and expectant fathers online revealed that fathers typically use forums such as Reddit as a platform to seek support from peers during difficult times. The discussion topics of *Pregnancy Complications* for expectant fathers and *Struggles* for current fathers were the largest discussion topics of each forum, with *Struggles* emerging as the largest discussion topic across both forums. These discussion topics also had the largest total word count and highest average word count per post, indicating that distressed fathers wrote comparatively longer posts and the online fatherhood community responded with detailed replies. These observations could suggest that social media sites such as Reddit may offer fathers some therapeutic benefits through the opportunity to write about and reflect on emotional experiences within the support network of an anonymous community. Quantitatively comparing the 2 forums indicated that distinctly different conversations occur before and after the birth of a child, suggesting that issues relevant to fathers antenatally are significantly different to postnatal issues.

Importantly, our findings reflect those observed in the wider literature exploring the transition to fatherhood, thereby supporting the validity of this novel sampling frame and analytic technique. We hypothesized that a range of discussion topics identified in the fatherhood transition literature would emerge within the Reddit fatherhood forums such as parenting practices, work-life balance issues, and spousal relationship issues. All these issues were observed within the forums but were grouped by the machine learning algorithm into broader categories. Discussions observed in our study are similar to that reported by StGeorge and Fletcher [22] in their support forum for fathers, with both *Announcements* and *Fathers’ Practices* identified as 2 of the primary discussion themes in both studies. Furthermore, Ammari et al [21] identified similar discussion topics in their analysis of mothers’ and fathers’ discussions on Reddit, including *Cheap Products* similar to our *Budgets and Purchases* category, *Sleep Training* similar to our *Sleep* category, and *Milestones* similar to our *Children’s Growth and Development* category*.* These themes appear to be common to the fatherhood experience, in that there will always be advice seeking, bonding, sharing of stories, and challenges in balancing work and family life.

However, there were some differences in the discussion topics observed in this study and that reported in previous research exploring the fatherhood transition in online forums. StGeorge and Fletcher [22] identified *Isolation* as an additional discussion topic that was not observed in our analysis. StGeorge and Fletcher [22] describe *Isolation* as the experience of fathers in being neglected from infant-parent welfare. Differences in the intent and userbase of the forums analyzed in the 2 studies could have resulted in different discussion topics. Specifically, as StGeorge and Fletcher’s [22] forum was created by a health care organization to support fathers across the fatherhood transition, fathers drawn to such a group might have been prepared to discuss how health care organizations currently neglect fathers in infant-parent welfare and how such organizations can provide better support to fathers. Furthermore, perhaps the clinical setting of the forum itself could contribute to fathers’ perceptions of isolation; while Reddit integrates discussions on fatherhood into a feed of discussions on any topic of interest, the creation of stand-alone websites for discussions of a single topic could itself be an isolating method of outreach to fathers. Finally, the missing theme from this study could reflect improvements in community discourse of and expectations about involved fathers over time. The changing nature of fatherhood and improvements in the father-inclusive practice of organizations could have helped to reduce the isolation experienced by fathers over the 8 years between the Web-based discussions of participants in StGeorge and Fletcher’s [22] study and this study.

Secondary contributions from this study are the ecologically valid social support techniques that were observed within online fatherhood communities, which may have important implications for the design of support interventions for fathers both in person and online. Fathers in both forums were observed to be using social support techniques reported in other studies, such as providing encouragement, confirming that issues or experiences are common and normal, reciprocal sharing of experiences, providing advice based on experience, sharing of information and resources, and the promotion of best practice or good examples of fatherhood practices [20]. Some additional social support techniques were also observed within the Reddit community, such as the importance of humor to build social connections and the use of Reddit’s *upvote* system to provide endorsement and support to original posts and comments. Community-building techniques were also demonstrated, such as the use of *Meet my Baby!* posts to introduce new users to the group, *Graduation* posts as a farewell message by users who were leaving the group, and holiday- or event-specific discussions to share a connection between the Web-based environment and real-world community events.

### Limitations

Importantly, the results of this study reflect the nature of the social media platform and community from which data were gathered. Reddit provides fathers with an anonymous platform to join a positive and supportive Web-based community of other men, with fatherhood discussions easily integrated into other discussion forums that fathers might subscribe to within the broader Reddit forum network. For researchers exploring Reddit data for fatherhood research, there are some important considerations to note. First, although Reddit is an internationally accessible website, it is likely to be biased to a Western experience of fatherhood, particularly toward North-American fathers. This is because of the site being hosted in North America, the predominant use of English across both r/Daddit and r/PreDaddit, and because of North America being the country of origin for the majority of Reddit users generally. Second, a potential limitation of Reddit is that it is difficult to determine how many passive users (commonly termed as *lurkers*) there are that may benefit from the discussions. Passive users do not actively participate in discussions via original posts or comments, making it difficult to calculate how many passive users are observing discussions and whether the discussions at hand are reflective of passive users’ experiences as well. Finally, the anonymous nature of Reddit means that the identities of users within the fatherhood forums are not verified. Although r/Daddit and r/PreDaddit are self-described as forums for current and expectant fathers, some users contributing to discussions may not actually be a father. Future research could consider comparing the discussions identified in this study with other pseudonymous or identifiable social media sites, such as Facebook or Twitter, where the identities of users can be verified and the nature of discussions are likely to differ, given the *real-world* relationships between users.

A further contribution of this study is the demonstration of applying *k*-means clustering to provide initial groupings of topics, which were further analyzed using traditional qualitative techniques. This demonstrated a new tool for researching fathers on the Web, complementing the Latent Dirichlet Allocation technique used by Ammari et al [21]. However, there are some limitations that are important to consider when using *k*-means. A common challenge in using *k*-means clustering is that it can also be difficult to identify the correct number of clusters within an unknown dataset (eg, the value of *k*), and the algorithm will produce the exact numbers of clusters denoted by *k—* even if that exact number of clusters does not exist within the dataset. In this study, we considered the results of several different values of *k* to determine which number of clusters produced the most semantically meaningful groups and then performed additional qualitative coding and analysis to uncover themes and discussion topics. Another characteristic of *k*-means is that each data point is grouped into a single cluster, whereas it is more realistic that discussion topics could comprise many subtopics. For example, in the combined groupings, we identified an entire category that was labeled *Sleep*, but we still identified some posts involving sleep in the *Struggles* category, albeit typically discussing sleep within a broader context of difficulties. Future studies could examine the effectiveness of different machine learning techniques for exploring fatherhood transitions using social media data. Finally, the size and unstructured nature of the dataset limited the opportunity for in-depth analysis of specific subtopics. Future research could consider delving further into specific topics either through text analysis techniques or through qualitative analysis using our clustered discussion themes as an initial framework.

### Conclusions

This study demonstrates a novel technique for exploring fathers’ experiences across the transition to parenthood by mapping the issues that men discuss with peers before and after childbirth. The discussion topics and social support techniques identified by this study can assist in the design of support programs and interventions for fathers. Importantly, using topic modeling of social media data offers unique clinical utility compared with traditional fatherhood research methods, as the discussions are current and timely and can be collected and analyzed at large scale in a short period.
